# What must not be named: Facts, emotions and coping strategies to enhance animal welfare in research

**DOI:** 10.1038/s44319-025-00429-1

**Published:** 2025-03-31

**Authors:** Katarina Riesner, Linda Hammerich, Denise Jahn, Agnes Ellinghaus, Dietrich Polenz, Lisa Grohmann, Juliane K Unger

**Affiliations:** 1 Taskforce Refinement, Charité – Univeristätsmedizin Berlin, Corporate Member of Freie Universität Berlin and Humboldt-Universitätsmedizin Berlin, Berlin, Germany; 2 Department of Hematology, Oncology and Tumorimmunology, Charité – Universitätsmedizin Berlin, Berlin, Germany; 3 Department of Hepatology and Gastroenterology, Charité – Universitätsmedizin Berlin, Berlin, Germany; 4 Department of Oral and Maxillofacial Surgery, Charité – Universitätsmedizin Berlin, Berlin, Germany; 5 Julius Wolff Institute, Charité – Universitätsmedizin Berlin, Berlin, Germany; 6 Department of Surgery, Experimental Surgery, Charité – Universitätsmedizin Berlin, Berlin, Germany; 7 Charité 3R, Charité – Universitätsmedizin Berlin, Berlin, Germany; 8 Research Facilities of Experimental Medicine, Charité – Universitätsmedizin Berlin, Berlin, Germany

**Keywords:** Economics, Law & Politics, Methods & Resources, Science Policy & Publishing

## Abstract

Scientists working with animals must maintain a delicate balance between conducting high-quality science and legal and ethical responsibilities. It is time to openly discuss their workload and emotional burden to improve both animal welfare and scientific quality in biomedical research.

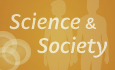

Animal studies continue to play a crucial role in biomedical research, but animal experimentation has always been a controversial topic. The societal debates about the ethics of using animals in research and animal wellbeing generally have led to increasingly strict regulations, such as gradual revisions of the European Directive 2010/63/EU on the protection of animals used for scientific purposes. Researchers working with animals must maintain a delicate balance between conducting necessary experiments and legal and regulatory requirements, which can compromise scientific validity, and increase the workload and administrative burden along with psychological pressure. These challenges underline the need for specialized animal research facilities and 3R centers that foster ethical awareness, provide institutional support, and encourage collaboration to produce high-quality research that addresses both animal welfare and contributes to scientific progress.

Moreover, scientists need to openly discuss the impact of increased regulations for scientific validity and reproducibility, their workload, and the emotional burden along with the improvements for animal welfare. An open debate about these issues would help to maintain society’s support for animal experiments in biomedical research and convince funders and politicians that achieving both high-quality research and animal welfare must be adequately financed and rewarded.

… scientists need to openly discuss the impact of increased regulations for scientific validity and reproducibility, their workload, and the emotional burden along with the improvements for animal welfare.

## Animal welfare regulations are hardly known or underrated by society and activists

While the requirements for animal welfare in research have increased globally, laws, regulatory processes and control mechanisms still vary to a large extend between jurisdictions. Some countries such as the USA rely on internal—but potentially biased and weaker— approval processes that take little time, whereas many European countries require approval and monitoring via internal animal-welfare bodies and external, mainly governmental, authorities. These regulations go beyond experimental conditions to cover animal housing and handling. Here, differences exist between individual countries with respect to structural details, duration of approval processes—from a few weeks in Austria to several months in Germany or the UK—and the tasks of the internal and external authorities, such as who monitors compliance with regulations.

The most important effects are the same: scientists and their institutional consultants for animal welfare must navigate an extensive review process involving government agencies and ethical committees to secure approval for each scientific project that uses animals. Applications require detailed information and thorough discussion regarding the research rationale and methods, the availability of alternative methods both for preparation and implementation of the project as well as ethical evaluation of potential harm for the animals.

The final approval constitutes a binding contract detailing every aspect of the scientific protocol between the scientist(s) and the authorities. Speaking for Germany, scientists are personally and legally responsible for strict compliance. Deviations from the approved protocols are punishable by fines or even prison sentences. The external governmental authorities are liable for their decisions to approve the requested protocols to the public and to third parties, notably animal rights organizations, who can and will sue them whenever they feel the approved experiments are in violation of the law. Even the smallest change in the protocol requires an additional internal and external review and approval. Continuous follow-up by institutional installed animal-welfare officers and the governmental authorities add to this time-consuming process.

Research institutes also experience a significant pressure to increase refinement and animal welfare beyond the actual approved experiments (Gouveia and Hurst, 2013; Hurst and West, 2010; Young et al, 2023); the EU Directive 2010/63/EU requires the implementation of refined handling measures via training programs for all species used in experiments. Unique for Germany, government authorities already integrated refinement requirements into the official approval process for research protocols, even for already approved and running research projects. These refinement are versatile and range from improved housing, training and habituation programs to experimental handling methods (https://www.3hs-initiative.co.uk/; https://nc3rs.org.uk/3rs-resources). Improved housing conditions include a number of enrichments, such as different types of houses, tunnels, levels, grids and other climbing opportunities as well as nesting materials and treats. Various European research institutes already provide more enriched and refined housing conditions than what is the norm internationally. Training programs to habituate animals to their handlers have been known for many years from medical care for pets or zoo animals and include stress-reducing methods to apply substances or picking-up mice via tunnels or cupped hands instead of picking them up by the tail.

Research institutes also experience a significant pressure to increase refinement and animal welfare beyond the actual approved experiments…

The high bureaucratic hurdles and regulations present both opportunities and challenges. While significant advances in animal welfare are being made and valuable refinement measures can and must be implemented, scientists and institutions face increased workload and responsibilities. Confronted with numerous changes and regulations, it is no surprise that scientists harbor substantial concerns about standardization, biological reproducibility and feasibility. The scientific community is actively discussing how to navigate these requirements, and a shift in mindset could foster better adaptation. Nonetheless, instead of viewing these measures as threats to scientific validity, we can see them as opportunities for improvement.

Confronted with numerous changes and regulations, it is no surprise that scientists harbor substantial concerns about standardization, biological reproducibility and feasibility.

## Accepting biological variation to enhance scientific meaningfulness

During the past 60 years, laboratory animal science has attempted to improve the reliability and reproducibility of experimental results by standardizing animal genetics and minimizing external variables. The idea behind this is that reducing environmental variability limits biological responses, thereby requiring fewer animals and boosting statistical power. Problems in terms of high data variation or in reproducing published methods in animals studies prompted even further reduction of environmental variables, leading to ever stricter animal-husbandry conditions. Yet, we continue to face a “crisis of reproducibility” as challenges in translating experimental findings from animals to humans persist (Baker, 2016).

It is time for a critical reevaluation of our current ‘reduce to maximum standardization’ mentality when it comes to husbandry and handling of animals. Today’s methods still give several relevant variables: size and materials of cages and their microclimates, watering systems with or without preservatives, different bedding and nesting materials; diets show a wide range in macronutrients and lack age adaption. Beyond the materials, we have an uncontrollable, unreducible variation regarding the people being involved in care, transport and experimentation. None of these factors are internationally standardized.

It is time for a critical reevaluation of our current ‘reduce to maximum standardization’ mentality when it comes to husbandry and handling of animals.

What Bruce Alexander and colleagues discussed already 40 years ago (Alexander et al, 1981) has now evolved into a new understanding of animals’ needs concerning their environmental exposure. Various behavioral studies show that animals exhibit individual reactions, learning curves and physiological responses even under minimal variation. The earlier ‘reduce to the max’ strategy did neither succeed in achieving ‘biological normality’ for animals, nor did it establish the standardization needed for reproducibility. Instead, it has led to artifacts, as standard housing is rather a model for the sedentary, crowded and distress-loaded pathology of modern human lifestyles (Alexander et al, 1981; Rojas-Carvajal et al, 2022).

Although it is frequently discussed that increasing cage enrichment may lead to higher data variability, several studies report comparable or even lower variation (Richter et al, 2009). Additionally, behavioral and neuroscience studies describe a refining impact of more enriched living conditions and improved stress procedures, for instance to avoid artificially induced, general pathophysiology such as depression (Ratuski and Weary, 2022; Wolfer et al, 2004). Increasing cage enrichment aims to provide more coping strategies for all kinds of stress and social interactions. This is assumed to standardize resilience in laboratory animals independent of their social rank or their personality, which in turn may be the reason for stable or reduced data variability as has been reported. A change in mentality by accepting the boundaries of standardization and recognizing the inherent biological variation can enhance the meaningfulness of experiments by acknowledging that some factors are uncontrollable, leading to more relevant and applicable findings.

## Fast developments in science are contrasted by slow improvements in animal welfare

In addition to animal welfare, improvements in study design and reporting have gained attention, leading to the 6Rs principle: replacement, reduction, refinement, reproducibility, robustness, and reporting (Russell and Burch, 1959; Strech and Dirnagl, 2019). Moreover, the introduction of new targets and research tools including shifting genetic backgrounds of inbred mice and genetically engineered animals, has resulted in a loss of direct comparability with older studies. While incorporating both sexes, different strains and mature or older animals is a positive development to address the “crisis of reproducibility”, any change in methods diminishes the comparability to previous trials, whether through improved anesthesia, analgesia, imaging in place of postmortems or other enhancements. It is therefore essential to conduct parallel control and treatment groups, as many factors cannot be consistently controlled over the years or due to changes in research facilities. Consequently, the necessary advancements in methods, such as training or the use of highly enriched cages, are acceptable as long as both control and treatment groups receive them equally and are thoroughly described and discussed regarding their potential impact (Ratuski and Weary, 2022).

Scientific progress relies on an open mindset and curiosity. Scientists are accustomed to implementing controls and regularly reevaluating their results with improved methods. This is contrasted by the seemingly slow improvements of animal welfare in the biomedical sciences. New refinement measures, such as training protocols, environmental enhancements and better application procedures, may reduce comparability with previous studies but can increase the scientific value of the findings.

Refinement helps to reduce unwanted stress in test animals, which in turn minimizes variability in individual reactions. This leads to more reliable data, which enhances reproducibility and, ultimately, better implementation of results. Additionally, for our experiments, a well-functioning immune system is crucial. The increased handling of animals through training protocols boosts antigen exposure, promoting the development of a mature immune system—something typically lacking in housing systems with individually ventilated cages that are designed for extreme hygiene but also limit the animals’ exposure to ubiquitous antigens (Bucher et al, 2019).

It is evident that we have never fully achieved, and likely never will achieve, complete comparability with past studies, as new methods, study designs and statistical approaches continue to evolve. When we focus on the biological benefits of improved animal wellbeing, the current challenges are simply part of science’s ongoing development. However, for institutions and researchers conducting animal experiments, the implementation of new welfare measures not only offers scientific benefits but also increases pressure from various sources: changing legal requirements, scientific competition, ethics and societal expectations. While this pressure can drive positive change, it may also create psychological barriers that hinder progress. In this way, emotions become factors we must consider in discussions on research quality (Pfister and Bohm, 2008).

When we focus on the biological benefits of improved animal wellbeing, the current challenges are simply part of science’s ongoing development.

## Training has benefits but significantly impacts on workload

The new required training protocols involve not only familiarizing the animal with the human experimenter, but it is also bidirectional training. During these training sessions, the handler gets to know the individual animal, which helps alleviate fears of bites or the animal escaping. This mutual understanding fosters trust and reduces stress for both parties. Our PhD students consistently report positive outcomes from the training, with the main drawback being the time it consumes. Contrary to our expectations, the training itself typically only takes a few minutes each day. However, it’s important to recognize that the time spent on organization, travel, and hygiene measures far exceeds the minutes required for the actual training. While we clearly see the benefits of the training, the additional time commitment significantly impacts workload, which should be taken into account when considering work schedules or future funding.

While we recognize the value of training protocols and strive to implement new refinement measures, we are often constrained by financial and organizational limits. For instance, a PhD student on a three-year contract simply cannot afford to spend six months or more establishing training programs or further refinement measures before beginning experiments. Even when an established training program exists, the time required to train all the animals involved in a project poses a significant burden for researchers, who also need to collect and analyze data for their actual studies. This issue could be addressed by hiring additional personnel, such as technicians or animal caretakers, to handle animal training. However, the question remains: who will cover those costs?

Germany’s academic and funding systems create a catch-22 situation. While funding agencies such as the German Research Foundation (DFG) require discussion of the 3Rs in research proposals (Forschung and Forschungsgemeinschaft, 2016), they often refer funding needs for core staff to host institutions, which typically lack the budgetary flexibility to accommodate additional positions or improved housing facilities and systems.

Moreover, the academic system’s emphasis on publishing for continued funding and job security adds pressure on young researchers to publish quickly and frequently, preferably in high-ranking journals. Unfortunately, refinement methods are often overlooked by journals. Although most journal guidelines ask for reporting animal-related procedures considering the ARRIVE or PREPARE guidelines, publications often still lack proper and complete reporting of this information as well as negative or null results (Hunniford et al, 2021; Sansom et al, 2024). This undermines the importance and scientific validity of adequate animal welfare measures. Making the situation even more challenging, standardized definitions are missing in the field of animal experimentation and terms like “enrichment” or “standard housing conditions” are differently interpreted world-wide (Cait et al, 2022).

## Am I able to withstand the mental strain?

Scientists performing animal experiments experience the dilemma of valuing an animal’s life, while also ending it. This statement may seem questionable and contradictory, but it reflects the inner and outer struggle we navigate in our daily work. We fully understand the sacrifices we are making and believe strongly in the importance of animal-based research for human health and society. Given the complexity of this sacrifice, we strive to ensure it is meaningful and take responsibility for our actions, a challenge that can be difficult to bear.

In light of recent findings regarding animals’ needs, species-specific behavior, and their complex capacity for suffering (Kahnau et al, 2020), those involved are beginning to doubt themselves even more than usual. These bioethical considerations further contribute to the mental load associated with rising bureaucratic and organizational demands, and funding challenges. Additionally, public perception often portrays scientists conducting animal experiments as “bad players”, failing to recognize the various animal welfare measures in place. While the push for improved animal welfare is important, it also places a heavy responsibility on individual researchers, leading to significant organizational and emotional burdens. To mitigate these challenges and counter feelings of losing control, effective strategies to develop solutions and a collaborative support system are essential.

… public perception often portrays scientists conducting animal experiments as “bad players”, failing to recognize the various animal welfare measures in place.

## 3R centers can serve as independent hubs for cooperation, networking, and support

As discussed above, refinement implementation means better animal welfare but comes at the cost of less standardization and an increased financial and mental burden. To overcome these unresolved challenges, a collaborative approach between different stakeholders is necessary. On the one hand, funders and journals need to recognize animal welfare for scientific validity by valuing it, insisting on its implementation and accepting above named boundaries along with increased funding for better animal housing and handling. On the other hand, supportive frameworks at scientific organizations are needed to allow the best possible refinement.

Establishing 3R centers as permanent structures in biomedical research institutions reflects a shift in mindset towards taking responsibility and prioritizing efforts to improving laboratory animal welfare and promoting non-animal experimentation (Harrison, 2024). Charité – Universitätsmedizin Berlin established its own 3R center in 2018 to advance the principles of the 3Rs (Replacement, Reduction, Refinement (Russell and Burch, 1959)) in biomedical research. Charité 3^R^ offers various initiatives in communication, education, support and funding (Retter et al, 2024) to enhance awareness and implementation of the 3Rs (https://charite3r.charite.de/en). Researchers are guided in using the most appropriate models in a scientifically robust and ethical manner, whether in vitro, in silico, or, when necessary, in vivo.

## Funding is key

Independent of any topic, progress always relies on resources, which in turn depend on funding. To support the implementation of the 3Rs in translational research models, Charité 3^R^ has developed a variety of internal funding lines. Roughly one-third of the total funding has been allocated to refinement initiatives, addressing an area often overlooked by traditional funding agencies. Another funding line, specifically available to staff in the animal housing facility, has facilitated the widespread adoption of new measures, such as stress-reducing handling techniques for mice and enhanced housing conditions. These initiatives have had a direct, positive impact on animal welfare at Charité.

Research shows that isolating animals from sensory stimuli, exploratory opportunities, social interactions, and physical activity leads to chronic stress. However, we often face the assumption that changing housing conditions could impair our scientific program. With funding from Charité 3^R^, we investigated the impact of enriched housing on our results. We compared different enrichment strategies, such as various cage types, designated playtime, and larger cages for bigger social groups. When taken to their playground, our rats eagerly leap into the adventure area, demonstrating trust that they will receive playtime and even volunteering to return to their smaller home cages. One part of our program has already yielded precise descriptions of our markers and comparability of results (Roschke et al, 2024), enabling us to proceed with enriched housing and make valuable observations to improve the interpretation of our study findings.

A different funding initiative supports core facilities to enhance their 3R-related services, focusing on systematic reviews of animal studies and imaging. These financial bridges, leading to successful follow-up funding, help maintain services that utilize highly specialized modern methods and ultimately improve animal welfare and scientific quality. Recognizing the need for sustainable expertise and infrastructure, further support was provided for our small-animal imaging center by establishing a professorship that simultaneously promotes 3 R research and education (EPIC3R).

## It’s not just about the money: it’s essential to support each other

Through various educational initiatives, Charité 3^R^ engages the research community’s diverse stakeholders. To educate the next generation of scientists, we offer interdisciplinary 3R training and collaborate with graduate and PhD programs at Charité and throughout Berlin. To encourage senior researchers to rethink their experimental procedures, Charité 3^R^ co-founded the “Taskforce Refinement”, which brings together experts from animal welfare officers to technicians to researchers, working collaboratively to promote the implementation of refinement practices at Charité. By meeting internally, we offer a confidential, non-competitive platform for professional and interpersonal exchange. Additionally, we created the “Refinement Sharepoint”, an internal hub that facilitates the free sharing of newly developed findings and methods. This hub provides access to short videos, protocols, seminars and original publications where applicable, making new knowledge easily accessible and engaging.

The exchange of information and new methods in a confidential environment enables progress even before publication and promoting animal welfare without compromising scientific success on the international stage in terms of potential publications or funding. In this supportive environment, researchers can confront discomforting feelings and channel their emotions as motivation for improvement. This mindset also led to the creation of hands-on medical training and refinement courses, where experienced scientists provide practical training to less-experienced colleagues, helping to spread enhanced animal welfare practices.

In this supportive environment, researchers can confront discomforting feelings and channel their emotions as motivation for improvement.

The experiences reported by scientists from their refinement approaches are both encouraging and motivating, helping to lower the perceived performance barrier for others and triggering a chain reaction of method adoption. By working together to improve fundamental methods rather than competing within the organization, more energy is conserved for the external competitive challenges, indirectly ‘repaying’ the resources committed to Charité 3^R^.

## Speak of the unspeakable instead of false arguments and denial

The challenges and hurdles discussed in this article are not unique to Charité — Universitätsmedizin Berlin; animal research institutions world-wide face similar situations daily. Still, significant parts of the scientific community resist implementing refinement measures, particularly cage enrichment and training protocols, arguing with the loss of standardization and reproducibility. This argument is no longer valid. Only by naming the unspeakable—such as emotional stress, lack of resources and priorities, and destructive competitive structures —we can develop effective solutions and implement new concepts.

Since the establishment of our infrastructure and measures, several improvements have been made at Charité. Working hand in hand with all involved parties—scientists, animal caretakers, technicians, veterinarians, experts in core facilities, institutional animal welfare bodies and dedicated funding programs—we can advance animal welfare in research. This comprehensive set of measures empowers those involved in animal experiments to realign their values and restore their ethical and emotional balance. It encourages the implementation of innovative approaches to address limited resources and fosters the joy of collaborative, trusting work, rather than wasting energy on outdated programs and internal competition. This effort leads to enhanced research quality and improved animal wellbeing. Ultimately, this approach reinstates trust and control for researchers, resulting in more robust outcomes and reliable models for translational research.

Enhancing animal welfare in research is a challenging endeavor requiring a step-by-step approach, whereby the scientific community, despite its diverse demands and opinions, works toward a common goal: achieving the highest quality in research to tackle pivotal biomedical questions. This should, in our opinion, be accompanied by robust outreach methods to educate the public on opportunities and challenges associated with implementing the 3Rs in research, as this holds the potential to induce an important mindset change. Awareness of available methods to increase animal welfare and the fact that they are already being used might increase support and recognition of research from the general public. This would in turn increase the pressure on authorities and funders to provide the necessary resources for 3Rs implementation, especially refinement measures. In addition, public recognition would provide a feeling of validation for scientists and further increase the motivation to implement 3R methods in their research. Thereby, the responsibility for 3R implementation would be shared by science and society and no longer carried by scientists alone.

## Supplementary information

Peer Review File
