# Supplementary material for: What must not be named: Facts, emotions and coping strategies to enhance animal welfare in research
Source: EMBO Rep. 2025 Mar 31;26(8):1929–34. doi: 10.1038/s44319-025-00429-1 (PMC12019234; doi:10.1038/s44319-025-00429-1)
Supplement: Supplementary file 1 — Peer Review File [file 44319_2025_429_MOESM1_ESM.pdf]

EMBO reports

What Must Not Be Named

Katarina Riesner, Linda Hammerich, Denise Jahn, Agnes Ellinghaus, Dietrich Polenz, Lisa Grohmann, and Juliane Unger

Corresponding author(s): Lisa Grohmann (lisa.grohmann@charite.de) , Juliane Unger (juliane.unger@charite.de)

Review Timeline:

Submission Date:	16th Dec 24
Accepted:	12th Mar 25

Editor: Holger Breithaupt

Transaction Report: This article was editorially reviewed.
